# Older Adults Vastly Overestimate Both HIV Acquisition Risk and HIV Prevalence in Rural South Africa

**DOI:** 10.1007/s10508-021-01982-1

**Published:** 2021-10-01

**Authors:** Eva van Empel, Rebecca A. de Vlieg, Livia Montana, F. Xavier Gómez-Olivé, Kathleen Kahn, Stephen Tollman, Lisa Berkman, Till W. Bärnighausen, Jennifer Manne-Goehler

**Affiliations:** 1 Harvard Center for Population and Development Studies, Harvard University, 9 Bow Street, Cambridge, MA 02138, USA; 2 Faculty of Health, Medicine and Life Sciences, Maastricht University, Maastricht, The Netherlands; 3 Medical Research Council/Wits Rural, Public Health and Health Transitions Research Unit, School of Public Health, University of the Witwatersrand, Johannesburg, Parktown, South Africa; 4 Department of Global Health and Population, Harvard T.H. Chan School of Public Health, Boston, MA, USA; 5 Africa Health Research Institute, Mtubatuba, South Africa; 6 Heidelberg Institute of Global Health, University of Heidelberg, Heidelberg, Germany; 7 Medical Practice Evaluation Center, Massachusetts General Hospital, Harvard Medical School, Boston, MA, USA; 8 Division of Infectious Diseases Brigham and Women’s Hospital, Harvard Medical School, Boston, MA, USA

**Keywords:** HIV, HIV risk perception, HIV transmission, HAALSI, Agincourt South Africa

## Abstract

Perceptions of HIV acquisition risk and prevalence shape sexual behavior in sub-Saharan Africa (SSA). We used data from the Health and Aging in Africa: A Longitudinal Study of an INDEPTH Community in South Africa baseline survey. Data were collected through home-based interviews of 5059 people ≥ 40 years old. We elicited information on perceived risk of HIV acquisition and HIV prevalence among adults ≥ 15 and ≥ 50 years old. We first describe these perceptions in key subgroups and then compared them to actual estimates for this cohort. We then evaluated the relationship between sociodemographic characteristics and accurate perceptions of prevalence in regression models. Finally, we explored differences in behavioral characteristics among those who overestimated risk compared to those who underestimated or accurately estimated risk. Compared to the actual HIV acquisition risk of < 1%, respondents vastly overestimated this risk: 35% (95% CI: 32–37) and 34% (95% CI: 32–36) for men and women, respectively. Respondents overestimated HIV prevalence at 53% (95% CI: 52–53) for those ≥ 15 years old and 48% (95% CI: 48–49) for those ≥ 50 years old. True values were less than half of these estimates. There were few significant associations between demographic characteristics and accuracy. Finally, high overestimators of HIV prevalence tested themselves less for HIV compared to mild overestimators and accurate reporters. More than 30 years into the HIV epidemic, older people in a community with hyperendemic HIV in SSA vastly overestimate both HIV acquisition risk and prevalence. These misperceptions may lead to fatalism and reduced motivation for prevention efforts, possibly explaining the continued high HIV incidence in this community.

## Introduction

Tremendous resources have been invested to educate populations in regions of high HIV prevalence, such as South Africa, about HIV risk and prevention. However, little data exist to assess perceptions of HIV acquisition risk and prevalence at the population level, especially among older adults. In order to prevent HIV in high-risk populations, it is critically important to understand current perceptions of HIV and potential knowledge deficits among people living in areas of high prevalence and design interventions to fill these gaps (Rosenberg et al., 2017; van Heerden et al., 2017; GHO, 2018).

Risk perception plays a crucial role in risk behavior (Brewer et al., 2004; Gerrard et al., 1993, 1996; Kalichman & Cain, 2005). Accurate risk perception may promote protective behavior, and underestimation of risk may lead to more risky behavior (Brewer et al., 2004; Kalichman & Cain, 2005). Moreover, accurate perceptions of HIV risk are necessary in shaping rational action, whereas inaccurate perceptions deprive individuals of agency (Akwara et al., 2003; Noroozinejad et al., 2013). Overestimation of HIV acquisition risk and HIV prevalence may engender fatalism, leading to greater risk-taking and less care-seeking (Hess & Mbavu, 2010; Sileo et al., 2019). In contrast, if HIV acquisition risk and HIV prevalence are underestimated, people may not sufficiently protect themselves against potential infection with HIV. Therefore, it is important that individuals have accurate perceptions about HIV acquisition risk and prevalence. However, perceptions of risk of acquisition and prevalence of HIV among heterosexual partners are unknown in many regions of high HIV prevalence, including South Africa.

Several studies in the U.S. have shown a wide variation in the understanding of HIV prevalence by the general population (Kalichman & Cain, 2005; Rosenberg et al., 2016; White & Stephenson, 2016). For instance, one study showed that people were able to estimate their local HIV prevalence and that perceptions of HIV prevalence can impact sexual risk behavior. In prior studies, underestimation of HIV prevalence has been associated with having multiple sexual partners, less HIV testing, more high-risk sexual behavior and higher rates of sexually transmitted diseases (Kalichman & Cain, 2005). Recent findings from the Health and Aging in Africa: Longitudinal Studies of an INDEPTH Community in South Africa (HAALSI) study report HIV prevalence of 23% among adults aged 40 years and above in 2014–2015 in Agincourt, South Africa, and a prevalence of 20% in adults aged 50 years and older (Gómez-Olivé et al., 2013; M. S. Rosenberg et al., 2017). As such, it is important to understand perceptions of prevalence among adults in South Africa in a setting where the burden of HIV is especially high, as they may be a potential area for programmatic intervention to reduce future transmission incidence (Rosenberg et al., 2017; van Heerden et al., 2017). Using data from the HAALSI baseline survey, we measured HIV acquisition risk and HIV prevalence perceptions among older adults living in a rural South African community with a very high prevalence of HIV. We compared these perceptions to the estimates of their true value and establish the extent to which the accuracy of these perceptions was a function of sociodemographic factors.

## Method

### Participants and Procedure

This study used data from the HAALSI baseline survey. HAALSI is a study of older adults that seeks to characterize cardiovascular disease, cognitive health, dementia and HIV (Gómez-Olivé et al., 2018). The study included men and women in rural South Africa that are 40 years and older. A total of 5059 people participated (2345 men (46.4%) and 2714 women (53.6%)) with an overall response rate of 85.9% (Gómez-Olivé et al., 2018). The HAALSI cohort is situated in the Agincourt health and socio-demographic surveillance system (HDSS), which annually updates social, demographic and health changes of 116,000 participants (Kahn et al., 2012). Data collection took place from November 2014 until November 2015. Inclusion criteria were being 40 years of age or older on July 1, 2014, and a resident in the Agincourt study area for at least one year before the HDSS update round in 2013 (Gómez-Olivé et al., 2018). Fieldworkers were residents in the study area who were locally trained to collect data at the household level. Responses were recorded via an individual computer-assisted personal interviews system and were checked internally to ensure data quality and completeness (Gómez-Olivé et al., 2018).

### Measures

Four questions about perceptions of HIV risk and prevalence were extracted from the survey. Questions regarding HIV risk perception included:

What do you think the probability is that a man would become infected with HIV from only one act of unprotected vaginal intercourse with an already infected woman? Probability a man would become infected = *1 in* …

What do you think the probability is that a woman would become infected with HIV from only one act of unprotected vaginal intercourse with an already infected man? Probability a woman would become infected = *1 in* …

In addition, there were two distinct questions asked about the perception of HIV prevalence, as follows:

Out of every 100 adults 15 years or older, how many do you think have been infected with HIV? Range 0–100. Out of every 100 adults 50 years or older, how many do you think have been infected with HIV? Range 0–100.

We abbreviate these questions as follows: (1) perceived acquisition risk for a man, (2) perceived acquisition risk for a woman, (3) prevalence ≥ 15 years, and (4) prevalence ≥ 50 years.

To determine accuracy of the perceptions, we defined a range of responses that may be considered plausible based on the current literature. Perceived prevalence was defined as accurate when reported in the range of 9.9–29.9% for the question “prevalence ≥ 15 years” (the actual percentage is 19.9) and 10–30% for “prevalence ≥ 50 years” (the actual percentage is 20.0) (Gómez-Olivé et al., 2013; Rosenberg et al., 2017; World Health Organization (WHO), 2019). The chosen range of accuracy for HIV acquisition risk perception was equal to the 95% confidence intervals (CIs) presented in a study by Boily et al. (2009), ranging from 0.09% to 2.70% (Patel et al., 2014), which corresponds to a participant response between 1 in 1111 and 1 in 37 for the questions acquisition risk for men and for women. The correlates of “accurate” perceptions were also explored in logistic regression analyses. This analysis had as an outcome a range of plausible or “accurate” answers as defined above.

### Statistical Analysis

We performed three analytical steps for each of the four questions of interest. First, we describe the sociodemographic and health characteristics of the overall cohort. We then display the distribution of estimates provided for all four metrics of interest and the percentage of people who reported “accurate” perceptions of acquisition risk and prevalence in the cohort overall, based on our given definitions. Next, we used multivariable logistic regression analysis to assess the relationship between accurate reporting of HIV prevalence and several key sociodemographic characteristics including age, sex, educational attainment, HIV status, household wealth and marital status. In a supplementary regression analysis, we assessed whether these same key sociodemographic factors were associated with accurate estimates of HIV acquisition risk. Finally, we explored the fatalism hypothesis by describing differences in the core demographic variables and health behaviors across groups defined by the participants’ accuracy in assessing HIV acquisition risk and prevalence. This analysis was performed in two ways. First, we compared these characteristics in those who underestimate HIV prevalence or HIV acquisition risk as compared to those who overestimate this risk. Second, we compared these characteristics across groups defined by the severity of overestimation (high overestimators, mild overestimators, accurate reporters). The health behaviors of interest for this analysis were two measures of self-reported sexual risk behavior, namely having sex without a condom with someone you know is HIV positive and having sex in exchange for money, goods or services, and one question about whether the participant had ever been tested for HIV.

Age was categorized into the following groups: “40–49 years,” “50–59 years,” “60–69 years,” “70–79 years” and “ ≥ 80 years.” Education categories were defined as follows: “no formal education,” “some primary (1–7 years),” “some secondary (8–11 years),” and “secondary or more (12 + years).” Household wealth was quantified according to quintiles of a household asset index, based on the methodology of Filmer and Pritchett (2001), with one representing the poorest and five the richest households. Household wealth was measured by questioning about residence and ownership of certain indicators, such as car or television (Geldsetzer et al., 2018). Dried bloodspots (DBS) were obtained through finger pricks of consenting participants. DBS were tested for HIV antibodies and viral load as explained in a prior study by Gómez-Olivé et al. (2018). In these analyses, HIV status was divided into two categories: “HIV negative” and “HIV positive.” Marital status was categorized as “never married,” “currently married or living with partner,” “separated,” “divorced,” and “widowed.” Finally, we also explored three self-reported behavioral variables: (1) ever being tested for HIV, (2) condom use with someone you know is HIV positive, and (3) having had sex in exchange for money, drugs, goods or services. Each of these questions was answered with a binary “yes” or “no.” Analyses were performed using the statistical program SPSS (IBM SPSS Statistics 25).

## Results

### Baseline Characteristics

The population of interest in this study was the subset of HAALSI participants who had responded to at least one of the four questions of interest. Of the 5059 HAALSI participants, 4276 (84.5%) responded to at least one of these four questions. Response rates for the four questions were 80.2% for the questions about acquisition risk for a man, acquisition risk for a woman and prevalence ≥ 15 years and 80.1% for prevalence ≥ 50 years (Table 1). We provide a summary of differences in demographic characteristics between responders and non-responders to each of these questions (Appendix Tables 6 and 7). Among all respondents, 45.7% were men, the mean age was 60.8 (± 12.6) years, and nearly half (42.7%) of the respondents had no formal education (Table 1). Of the 3836 (89.7%) participants who consented to DBS HIV testing, 2948 (76.9%) tested negative, while 888 (23.1%) tested positive for HIV (Table 1).

### Perceptions of HIV Acquisition Risk

**Fig. 1:**
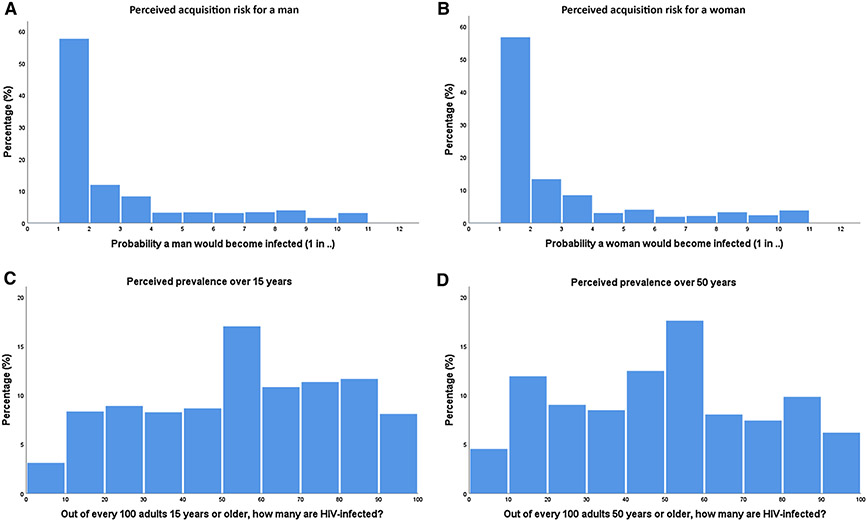
Histograms depicting the variation in perceptions of **a** per sex act acquisition risk for a man having vaginal intercourse with a woman living with HIV, **b** per sex act acquisition risk for a woman having vaginal intercourse with a man living with HIV, **c** prevalence of HIV in a population of 15 years or older and **d** prevalence of HIV in a population of 50 years or older

The mean perceived risk for a man becoming infected after one sex act with a woman living with HIV was 1 in 2.8 (± 5.6), which corresponds to 35.2%. For a woman, this number was slightly lower, with a mean perceived risk of 1 in 2.9 (± 5.0), corresponding to 34.2% (Table 2). Histograms (Fig. 1a, b) display distributions of these values and demonstrate that people from this South African cohort most frequently estimate the risk of acquiring HIV from one sex act to be between 1 in 1 and 1 in 10, for both a man (99.3%) and a woman (98.8%), with the most frequently stated risk being 1 in 1 (57.7% for a man, 56.6% for a woman). Those who did not accurately estimate HIV acquisition risk all overestimated the risk of acquiring HIV.

### Perceptions of HIV Prevalence

HAALSI participants reported that they perceived HIV prevalence among people aged 15 or older to be 52.7 in 100 (± 26.7). Among people aged 50 or older, the perceived prevalence reported by HAALSI participants was 48.1 in 100 (±27.4) (Table 2). Histograms (Fig. 1c, d) of the responses for both ≥ 15 and ≥ 50 years old show high variability, clearly demonstrating this population’s limited understanding of the true HIV prevalence.

### Evaluating the Association Between Sociodemographic Characteristics and Perceptional Accuracy

No significant differences were found in perceptions of HIV acquisition risk and prevalence by sex, age, education and HIV status. Moreover, univariable and multivariable logistic regression analyses likewise uncovered no significant differences in the unadjusted or adjusted association between perceptional accuracy and sex, education, HIV status and household wealth (Table 3, Appendix Table 8). However, older people appeared to be significantly better at accurately estimating the HIV prevalence among people aged 50 or older (*p* = 0.04). The odds ratios (ORs) that described accurate reporting of HIV prevalence in this group were 1.26 (*p* = 0.05) for “50–59 years,” 1.41 (*p* < 0.01) for “60–69 years,” and 1.33 (*p* = 0.05) for “70–79 years,” compared to people aged 40–49 years (Table 3). This indicates that the HAALSI cohort members have a slightly more accurate perception of HIV prevalence in their own demographic group than for the population overall. Moreover, the regression analysis showed that divorced people were less likely to accurately estimate the HIV prevalence among people over 15 years (OR 0.36, *p* = 0.01) and 50 (OR 0.56, *p* = 0.03) years old, with the reference group being people who were never married. People who had never been tested for HIV were less likely to accurately estimate the HIV prevalence both among those 15 years or older (OR 0.48, *p* < 0.01) and among those 50 years or older (OR 0.75, *p* < 0.01), compared to those who had ever been tested for HIV. Finally, we found no relationship between our self-reported measures of sexual risk behavior and the outcomes of interest (Table 3, Appendix Table 8).

### Exploring the Fatalism Hypothesis

There were few people who underestimated HIV prevalence over 15 years (*n* = 126) and 50 years (*n* = 183) old. Therefore, the vast majority of inaccurate reports were overestimates. We performed a subanalysis to examine differences in sociodemographic and health behavior characteristics among people who overestimated HIV prevalence compared to those who estimated accurately (Table 4). We found accurate reporters were slightly wealthier compared to those who overestimated HIV prevalence in both age groups (Table 4). Furthermore, we found more women who overestimated HIV prevalence than accurately estimated (54.6% versus 50.1%, *p* = 0.03) (Table 4). Though very few people underestimated HIV prevalence, we performed a second subanalysis to compare characteristics of those who under- and overestimate risk and prevalence (Appendix Table 9). In this supplemental analysis, we found that overestimators had greater educational attainment and were wealthier than people who underestimated HIV prevalence over 15 years old (Appendix Table 9).

In exploring the fatalism theory, we found no significant differences in sexual risk behavior for those who overestimate HIV acquisition risk compared to those who accurately do so. However, we found a significant difference in HIV testing behavior between those who overestimate HIV prevalence in people over 15 and 50 years old and those who accurately do so (Table 5). These results show that a lower proportion of people who provide high overestimates of HIV prevalence have ever been tested for HIV. However, we found no significant differences in responses to the sexual behavior questions between these two groups.

We also evaluated the relationship between overestimation of both HIV prevalence and acquisition risk and participant characteristics (Appendix Tables 10 and 11). In this analysis, we found that high overestimators of HIV prevalence in adults over 50 years of age were less wealthy than mild overestimators and accurate reporters (Appendix Table 10). Finally, we found no meaningful differences in participant characteristics and the degree of overestimating HIV acquisition risk (Appendix Table 11).

## Discussion

This study explored perceptions of HIV acquisition risk and prevalence among older adults in rural South Africa. Participants provided a very wide range of estimates for both risk and prevalence, and overall low rates of accuracy about these core principles related to the HIV epidemic. When considering an “actual” risk of acquiring HIV from one heterosexual sex act with a man or woman living with HIV being 1 in 1111 to 1 in 37, we found that most older adults overestimated this risk, providing frequencies from 1 in 1 to 1 in 10. In contrast, there was no obvious pattern in the perceived prevalence and few participants gave accurate estimates of this key parameter.

Accurately estimating HIV acquisition risk and prevalence was not associated with sex, education level, HIV status, household wealth or two measures of sexual risk behavior—condom use and sex in exchange for money, drugs, foods or services. However, older adults were more likely to estimate the prevalence in their own age group with greater accuracy. There were very few people who estimated HIV acquisition risk accurately, limiting our ability to draw definitive conclusions from these questions. The implications of these findings suggest that there are few sociodemographic factors that predict whether a person can accurately estimate HIV prevalence. In particular, it appears that many people believe the risk and prevalence are extremely high. We found that high overestimators of HIV prevalence were also less likely to have ever been tested for HIV, suggesting that fatalism might be present in this population.

Explanations for this wide distribution in the perception of HIV risk and prevalence might be that, despite awareness and educational efforts that have been part of prevention strategies, a knowledge gap still exists about HIV in this high-prevalence community. This could be due to a relatively minimal emphasis on older adults in preventive educational interventions, as these programs tend to focus more on younger people (Mills et al., 2011, 2012; Negin et al., 2012; Rammohan & Awofeso, 2010). The older population seems to be a neglected group in terms of education and awareness, which is supported by the finding that they know little about HIV acquisition risk and prevalence. However, previous research has also shown that only 34% of younger adults (under the age of 25 years) have an accurate understanding of HIV acquisition and prevention despite educational efforts (UNFPA, 2015), though in our older cohort the overall accuracy was even lower at only 11.6%. It is likely that our aging cohort received much less education about HIV and thus has a more limited understanding of the disease. Another possible explanation is that, given the stigma associated with HIV infection, people do not share their HIV status in social circles, leading to further misconceptions about the prevalence and risk of acquiring HIV in this context (Parker & Aggleton, 2003; Sandelowski et al., 2004; Treves-Kagan et al., 2017). An interesting finding is that accurately estimating acquisition risk and prevalence seem to have no significant sociodemographic predictors, except for the relationship between older age and the increased accuracy of prevalence estimation in older adults, as mentioned earlier. In particular, we hypothesized that education might be of importance in estimating risk and prevalence correctly. However, the “accurate” group had a small sample size and little variability in educational attainment generally, with 78.3% of participants having an education level below secondary school.

Several prior studies have investigated perceptions of HIV risk. However, many of these studies have focused on perceptions of lifetime risk of acquiring HIV instead of the risk of contracting HIV from one sex act (Chard et al., 2017; Clifton et al., 2016; Price et al., 2018). To the best of our knowledge, this is the first study to assess the understanding of acquisition risk in an older, rural South African population. One study that did investigate the perception of per sex act HIV acquisition risk was undertaken in the U.S. among men who have sex with men (MSM). This study demonstrated substantial misperceptions of risk, with most participants overestimating the risk of acquiring HIV in a single sex act with an infected partner (Belcher et al., 2005). Our study substantiates this existing misperception of HIV risk, now confirmed in an older population in South Africa.

A study in the U.S. investigated perceptions of prevalence of sexually transmitted infections (STIs), with the main focus being HIV, in men and women (Kalichman & Cain, 2005). Results showed that people were capable of accurately estimating the HIV prevalence in their hometown. However, people who did not estimate prevalence correctly were likely to overestimate the number of people living with HIV (Kalichman & Cain, 2005). This overestimation of prevalence is similar to our findings. However, the finding that people were able to accurately estimate the HIV prevalence differs from the findings in this study. A possible reason for this discrepancy is the context and specifically the greater availability of health education in the U.S. Moreover, the participants in this study were older South Africans, who grew up under the apartheid system where the educational quality in schools was lower for the black South African population (McKeever, 2017).

This study has important implications for understanding and potentially intervening to alter sexual risk behavior at the population level. Specifically, without accurate perceptions of risk, individuals lack agency to make the best choices regarding sexual risk-taking and HIV care-seeking (Akwara et al., 2003; Noroozinejad et al., 2013). Given the dramatic overestimations of both HIV acquisition risk and prevalence in this population, there is a reason to be concerned that fatalism may be deeply rooted among many in this community (Hess & Mbavu, 2010; Sileo et al., 2019). We find preliminary evidence this may be the case in our context because our results show people who were high overestimators of the HIV prevalence were less likely to test themselves for HIV. Individuals may not feel the need to protect themselves, because they perceive that they will acquire HIV regardless (Hess & Mbavu, 2010; Sileo et al., 2019; Sterck, 2013). Prior research has shown that awareness of high HIV prevalence and difficulties in consistent condom use might contribute to a sense of fatalism regarding HIV protection (Meyer-Weitz, 2005). Some might think that overestimation of HIV risk at the population is good because people will then take less risk. However, individuals may lack information about preventing HIV transmission and thus their fatalism is more plausible (Hess & Mbavu, 2010; Sileo et al., 2019; Sterck, 2013). Moreover, if there were strongly deterrent effects of these misperceptions, one would not expect such an extremely high HIV prevalence in this community. However, more research is needed to understand how perceptions of risk shape individual behavior. Regardless of the impact of these beliefs on behavior, it would be perverse to allow individuals to remain misinformed in order to manipulate their health behaviors.

In addition to overestimation of acquisition risk, we also find a high degree of uncertainty about prevalence at the community level. This is an important, distinct concern. This community-level uncertainty could be psychologically distressing, and it could also have a range of complex behavioral effects when people meet (e.g., in sexual encounters) who hold very different beliefs about transmission risk and the probability that a community member from the opposite sex is HIV positive (Halkitis et al., 2004; Kalichman et al., 2006). In order to equalize these perceptions about HIV acquisition risk and prevalence among this population, changes are needed to existing HIV prevention strategies. Potential ideas to improve education and intervention campaigns in the future include (1) more direct engagement with the aging community, (2) stronger social marketing campaigns with targeted, accurate messages, and (3) ensuring that health workers and traditional leaders are informed about these realities. As such, it is important to increase hope that the HIV epidemic can be controlled in this population. This can be achieved by informing this population of an 80% chance of testing negative for HIV and educating them about the transmission rate, which in the age of “undetectable equals untransmittable” is considered to be zero when a person is virally suppressed on ART (Barroso et al., 2000; Cohen et al., 2016; Rodger et al., 2016).

This study had several limitations. First, the perceptions of risk and prevalence were coded into binary outcomes (accurate and not accurate) and a range was chosen to determine accuracy. This range was chosen based on the actual biological risk and estimated prevalence and what seemed to be a plausible range around the known estimate. Hence, these ranges were partially subjective. Furthermore, the state of infection can influence acquisition risk, as is known that acquisition risk during acute infection increases infectious potential by 26-fold (Hollingsworth et al., 2008; Miller et al., 2010), whereas acquisition risk when having sex with a virally suppressed person living with HIV is close to zero (Barroso et al., 2000; Cohen et al., 2016; Rodger et al., 2016). The survey did not specifically discuss acquisition risk in specific stages of disease, and thus, there may have been ambiguity on the part of the respondents. Another limitation of this study is that the estimates of HIV acquisition risk were framed only in terms of unprotected sex. This overestimation of the risk of unprotected sex may or may not be extrapolated to people’s perceptions of the risk of HIV acquisition during sex with a condom. Moreover, the questions about acquisition risk were asked in the form of a probability; although introductory text^1^ was provided, this still had the potential to confuse respondents, especially those with low levels of education. Finally, we were not able to measure all potentially relevant factors, such as loss of a spouse to HIV or the influence of younger adults in the household on risk perceptions. As such, this study may be subject to residual confounding due to unobserved factors.

## Conclusion

In conclusion, 30 years into the HIV epidemic there still are substantial misperceptions and uncertainty about HIV acquisition risk and prevalence among this older South African cohort. This might in fact be one of the deeper, underlying drivers of the continued spread of this disease in sub-Saharan Africa, especially in this age group. HIV education and information in this population remain insufficient. Without better understanding, individuals may be deprived of agency in making decisions about sexual risk-taking and HIV care-seeking and, if overestimating risk, may also espouse a fatalistic attitude toward prevention of this disease. Expanded and improved education and information campaigns are urgently needed to ensure that older adults have correct perceptions about key aspects of their HIV risk.

## Figures and Tables

**Table 1:** Baseline characteristics of the HAALSI participants who responded to at least one of the four questions about perceptions of HIV acquisition risk and HIV prevalence

	N	% of population
*Sex*		
Men	1954	45.7%
Women	2322	54.3%
*Age groups*		
40–49	823	19.2%
50–59	1236	28.9%
60–69	1141	26.7%
70–79	701	16.4%
≥80	375	8.8%
*Education*		
No formal education	1822	42.7%
Some primary (1–7 years)	1520	35.6%
Some secondary (8–11 years)	525	12.3%
Secondary or more (12 + years)	403	9.4%
*HIV status* ^a^		
HIV−	2948	76.9%
HIV +	888	23.1%
*Household wealth index*		
Quintile 1 (poorest)	855	20.0%
Quintile 2	846	19.8%
Quintile 3	850	19.9%
Quintile 4	849	19.9%
Quintile 5 (richest)	876	20.5%
*Perceived acquisition risk for man* ^b^		
Estimated accurately	13	0.3%
Estimated not accurately	4042	99.7%
*Perceived acquisition risk for woman* ^c^		
Estimated accurately	16	0.4%
Estimated not accurately	4042	99.6%
*Perceived prevalence ≥15 years* ^d^		
Estimated accurately	711	17.5%
Estimated not accurately	3347	82.5%
*Perceived prevalence ≥50 years* ^e^		
Estimated accurately	1147	28.3%
Estimated not accurately	2904	71.7%
Total number of respondents	4276	

a Total number of participants who consented to DBS = 3836 (89.7%); not consented to DBS = 440 (10.3%)

b Total number of responses = 4055 (80.2%); missing responses = 1004 (19.8%)

c Total number of responses = 4058 (80.2%); missing responses = 1001 (19.8%)

d Total number of responses = 4058 (80.2%); missing responses = 1001 (19.8%)

e Total number of responses = 4051 (80.1%); missing responses = 1008 (19.9%)

**Table 2:** Distributions of perceived acquisition risk and prevalence of acquiring HIV

	Mean	Mean expressed in percentages	95% Confidence Interval	Standard deviation	Range (min–max)	Median	Interquartile range
Perceived acquisition risk for a man	1 in 2.8	35.2%	32.4–36.8	5.6	0–222	1	1–3
Perceived acquisition risk for a woman	1 in 2.9	34.2%	32.2–35.7	5.0	0–100	1	1–3
Perceived prevalence ≥ 15 years	52.7 in 100	52.7%	51.7–53.4	26.7	0–100	50	30–75
Perceived prevalence ≥ 50 years	48.1 in 100	48.1%	47.7–49.4	27.4	0–100	50	25–70

**Table 3:** Multivariate logistic regression models to examine the association between perceptional accuracy of HIV prevalence and sociodemographic covariates

Covariate	Accurate prevalence estimate ≥ 15 years				Accurate prevalence estimate ≥ 50 years			
	Multivariate				Multivariate			
	OR (95% CI)	*p* value	OR (95% CI)	*p* value	OR (95% CI)	*p* value	OR (95% CI)	*p* value
*Sex*		.28		0.27		.43		.58
Men	REF		REF		REF		REF	
Women	0.90 (0.74–1.09)	.28	0.90 (0.74–1.09)	0.27	1.07 (0.91–1.25)	.43	1.05 (0.89–1.23)	.58
*Age groups*		.14		0.01*		.04*		.03*
40–49	REF		REF		REF		REF	
50–59	1.33 (1.01–1.75)	.04*	1.43 (1.08–1.91)	.01*	1.26 (1.00–1.58)	.05*	1.27 (1.01–1.60)	.04*
60–69	1.48 (1.10–1.99)	.01*	1.70 (1.25–2.31)	< .01**	1.41 (1.11–1.80)	< .01**	1.46 (1.14–1.87)	< .01**
70–79	1.33 (.94–1.87)	.11	1.62 (1.13–2.31)	< .01**	1.33 (1.00–1.76)	.05*	1.39 (1.04–1.86)	.02*
≥ 80	1.43 (.95–2.15)	.09	1.86 (1.21–2.86)	< .01**	1.06 (.74–1.50)	.77	1.12 (.78–1.61)	.54
*Education level*		.67		.74		.81		.85
No formal education	REF		REF		REF		REF	
Some primary	1.08 (.87–1.33)	.50	1.01 (.81–1.26)	.92	1.00 (.84–1.19)	.97	.98 (.82–1.17)	.79
Some secondary	1.18 (.87–1.61)	.28	1.14 (.84–1.57)	.40	1.00 (.77–1.30)	1.00	1.01 (.78–1.32)	.92
Secondary or more	1.21 (.84–1.74)	.31	1.18 (.81–1.72)	.39	1.14 (.85–1.54)	.39	1.11 (.82–1.51)	.50
*HIV status*		.96		.72		.68		.95
HIV−	REF		REF		REF		REF	
HIV +	1.01 (.82–1.23)	.96	1.04 (.84–1.28)	.72	.96 (.81–1.15)	.68	1.01 (.84–1.20)	.95
*Household wealth index*		.04*		.03*		.09		.15
Quintile 1 (poorest)	REF		REF		REF		REF	
Quintile 2	.79 (.59–1.07)	.12	.79 (.58–1.08)	.14	1.09 (.86–1.38)	.49	1.10 (.86–1.41)	.45
Quintile 3	1.05 (.79–1.40)	.73	1.05 (.78–1.41)	.76	1.33 (1.05–1.68)	.02*	1.34 (1.05–1.71)	.02*
Quintile 4	1.26 (.95–1.67)	.11	1.30 (.97–1.74)	.08	1.18 (.92–1.50)	.19	1.20 (.94–1.54)	.15
Quintile 5 (richest)	1.14 (.85–1.53)	.40	1.08 (.79–1.47)	.62	1.35 (1.05–1.73)	.02*	1.29 (.99–1.67)	.06
*Marital status*		.00**		< .01**		.03*		.05
Never married	REF		REF		REF		REF	
Currently married/living with partner	1.18 (.77–1.82)	.46	1.26 (.79–2.00)	.34	1.01 (.73–1.43)	.90	1.02 (0.72–1.46)	.89
Separated/deserted	1.98 (1.21–3.23)	.00**	2.02 (1.20–3.41)	< .01**	1.24 (.83–1.86)	.29	1.18 (.78–1.79)	.44
Divorced	.36 (.16–.79)	.01*	.35 (.15–.81)	.01*	.56 (.34–.94)	.03*	.57 (.33–.96)	.04*
Widowed	1.26 (.79–1.99)	.33	1.26 (.77–2.05)	.36	.96 (.67–1.38)	.83	.95 (.65–1.38)	.78
*Ever tested for HIV*				< .01**				< .01**
Yes	—	--	REF		--	--	REF	
No	--	--	.48 (.39–.59)	< .01**	--	--	.75 (.64–.88)	< .01**

* *p* < .05

** *p* < .01

**Table 4:** Differences in participant characteristics between accurate reporting versus overestimates of the HIV prevalence among people 15 and 50 years or older

	HIV prevalence estimate 15 ≥ years					HIV prevalence estimate 50 ≥ years				
	Accurate		Overestimates		Chi-square	Accurate		Overestimates		Chi-square
	N	%	N	%	*p*-value	N	%	N	%	*p*-value
*Sex*					.03*					.90
Men	349	49.9%	1469	45.4%		530	46.2%	1251	46.0%	
Women	350	50.1%	1764	54.6%		617	53.8%	1470	54.0%	
*Age groups*					.12					.06
40–49	117	16.7%	651	20.1%		204	17.8%	553	20.3%	
50–59	206	29.5%	957	29.6%		337	29.4%	809	29.7%	
60–69	210	30.0%	837	25.9%		334	29.1%	688	25.3%	
70–79	109	15.6%	521	16.1%		189	16.5%	437	16.1%	
≥ 80	57	8.2%	521	8.3%		83	7.2%	234	8.6%	
*Education*					.77					.75
No formal education	274	39.2%	1334	41.3%		467	40.7%	1123	41.3%	
Some primary (1–7 years)	264	37.8%	1165	36.1%		416	36.3%	994	36.6%	
Some secondary (8–11 years)	91	13.0%	409	12.7%		144	12.6%	346	12.7%	
Secondary or more (12 + years)	70	10.0%	320	9.9%		120	10.5%	253	9.3%	
*HIV status*					.94					.41
HIV−	485	76.7%	2230	76.6%		801	77.3%	1865	76.0%	
HIV +	147	23.3%	681	23.4%		235	22.7%	588	24.0%	
*Household wealth index*					.05*					.03*
Quintile 1 (poorest)	134	19.2%	637	19.7%		200	17.4%	564	20.7%	
Quintile 2	110	15.7%	659	20.4%		215	18.7%	546	20.1%	
Quintile 3	141	20.2%	630	19.5%		244	21.3%	521	19.1%	
Quintile 4	159	22.7%	643	19.9%		225	19.6%	551	20.2%	
Quintile 5 (richest)	155	22.2%	664	20.5%		263	22.9%	539	19.8%	
*Marital status*					< .01**					.01*
Never married	33	4.7%	185	5.7%		62	5.4%	154	5.7%	
Currently married/living with partner	373	53.4%	1688	52.3%		618	53.9%	1410	51.9%	
Separated/deserted	83	11.9%	250	7.7%		108	9.4%	220	8.1%	
Divorced	11	1.6%	168	5.2%		33	2.9%	144	5.3%	
Widowed	199	28.5%	939	29.1%		326	28.4%	790	29.1%	
Total number of respondents	711		3233			1147		2721		

* *p* < .05.

** *p* < .01

**Table 5:** Differences in sexual risk behavior and HIV testing behavior between accurate reporters, mild overestimators and high overestimators of the HIV prevalence over 15 and 50 years old

	HIV prevalence estimate 15 ≥ years							HIV prevalence estimate 50 ≥ years						
	High overestimators^a^		Mild overestimators^b^		Accurate reporters^c^		Chi-square	High overestimators^a^		Mild overestimators^b^		Accurate reporters^c^		Chi-square
	N	%	N	%	N	%	*p*-value	N	%	N	%	N	%	*p*-value
*Ever sex without condom with someone HIV positive*							.45							.45
Yes	33	1.8%	21	1.6%	16	2.4%		33	1.8%	21	1.6%	16	2.4%	
No	1806	98.2%	1316	98.4%	659	97.6%		1806	98.2%	1316	98.4%	659	97.6%	
*Sex for money*							.93							.93
Yes	8	.4%	7	.5%	3	.4%		8	.4%	7	.5%	3	.4%	
No	1844	99.6%	1344	99.5%	688	99.6%		1844	99.6%	1344	99.5%	688	99.6%	
*Ever tested for HIV*							< .01**							< .01**
Yes	1163	62.5%	879	64.2%	543	78.5%		1163	62.5%	879	64.2%	543	78.5%	
No	697	37.5%	490	35.8%	149	21.5%		697	37.5%	490	35.8%	149	21.5%	
Total number of respondents	1861		1372		699			1861		1372		699		

* *p* <.05

** *p* <.01

a High overestimates were defined as people who estimated HIV prevalence to be 57.0% or higher

b Mild overestimates were defined as people who estimated HIV prevalence between 29.9% and 57.0% for prevalence ≥ 15 years old and between 30.0% and 57.0% for prevalence ≥50 years old

c Accurate reporters were defined as people who estimated HIV prevalence between 9.9% and 29.9% for prevalence≥ 15 years old and between 10.0% and 30.0% for prevalence≥ 50 years old

## Data Availability

Data are (partially) publicly available on the HAALSI Web site: https://haalsi.org/data. Codes are available from the corresponding author upon reasonable request.

## Footnotes

1 Introductory text: “HIV can be transmitted when an HIV-infected person has sex with an HIV-uninfected person. However, not every sex act between an HIV-infected and an HIV-uninfected person leads to an HIV infection. In the following questions, you will be asked to estimate the probability that a sex act leads to an HIV transmission. As an example of how we will ask you to answer this question, please consider the following example: “The probability of winning the lottery is 1 in ____” (the true probability of winning the lottery is somewhere around 1 in 10 million).”

## Appendix

See Tables 6, 7, 8, 9, 10, and 11.

**Table 6:** Independent t-test (age) and Pearson chi-square (others) for investigating differences in baseline characteristics between responders and non-responders to the two questions about “HIV acquisition risk”

	Responders (n)	%	Non-responders (n)	%	Chi-square	*p*-value
HIV acquisition risk for man						
*Sex*					1.22	.27
Men	1864	46.0%	481	47.9%		
Women	2191	54.0%	523	52.1%		
*Education*					75.70	<.01**
No formal education	1735	42.8%	571	57.6%		
Some primary	1431	35.3%	285	28.8%		
Some secondary	492	12.1%	82	8.3%		
Secondary or more	393	9.7%	53	5.3%		
*HIV status*					3.25	.07
HIV−	2793	76.5%	719	79.3%		
HIV+	860	23.5%	188	20.7%		
*Household wealth index*					4.94	.29
Quintile 1 (poorest)	817	20.1%	229	22.8%		
Quintile 2	814	20.1%	187	18.6%		
Quintile 3	793	19.6%	198	19.7%		
Quintile 4	804	19.8%	203	20.2%		
Quintile 5 (richest)	827	20.4%	187	18.6%		
	Mean±SD		Mean±SD		*t*-value	*p*-value
Age	60.7±12.6		66.0±14.1		10.94	<.01**
	Responders (n)	%	Non-responders (n)	%	Chi-square	*p*-value
HIV acquisition risk for woman						
*Sex*					2.43	.12
Men	1859	45.8%	486	48.6%		
Women	2199	54.2%	515	51.4%		
*Education*						
No formal education	1736	42.8%	570	57.7%	74.91	<.01**
Some primary	1435	35.4%	281	28.4%		
Some secondary	492	12.1%	82	8.3%		
Secondary or more	391	9.6%	55	5.6%		
*HIV status*					3.18	.07
HIV−	2794	76.5%	718	79.2%		
HIV +	860	23.5%	188	20.8%		
*Household wealth index*					3.93	.42
Quintile 1 (poorest)	818	20.2%	228	22.8%		
Quintile 2	811	20.0%	190	19.0%		
Quintile 3	795	19.6%	196	19.6%		
Quintile 4	809	19.9%	198	19.8%		
Quintile 5 (richest)	825	20.3%	189	18.9%		
	Mean±SD		Mean±SD		*t*-value	*p*-value
Age	60.7±12.6		66.0±14.2		10.90	<.01**

* *p* < .05.

** *p* < .01

**Table 7:** Independent *t*-test (age) and Pearson chi-square (others) for investigating differences in baseline characteristics between responders and non-responders to the two questions about “HIV prevalence”

	Responders (n)	%	Non-responders (n)	%	Chi-square	*p*-value
Perceived prevalence ≥ 15 years						
*Sex*					.18	.67
Men	1875	46.2%	470	47.0%		
Women	2183	53.8%	531	53.0%		
*Education*					159.32	< .01**
No formal education	1680	41.5%	626	63.3%		
Some primary	1463	36.1%	253	25.6%		
Some secondary	514	12.7%	60	6.1%		
Secondary or more	396	9.8%	50	5.1%		
*HIV status*					1.50	.22
HIV−	2805	76.6%	707	78.6%		
HIV +	855	23.4%	193	21.4%		
*Household wealth index*					13.96	< .01**
Quintile 1 (poorest)	801	19.7%	245	24.5%		
Quintile 2	799	19.7%	202	20.2%		
Quintile 3	803	19.8%	188	18.8%		
Quintile 4	815	20.1%	192	19.2%		
Quintile 5(richest)	840	20.7%	174	17.4%		
	Mean ± SD		Mean ± SD		*t*-value	*p*-value
Age	60.6 ± 12.5		66.6 ± 14.2		12.31	< .01**
	Responders (n)	%	Non-responders (n)	%	Chi square	*p*-value
Perceived prevalence ≥ 50 years						
*Sex*	1873	46.2%	472	46.8%	.11	.74
Men	2178	53.8%	536	53.2%		
Women						
*Education*					156.22	< .01**
No formal education	1678	41.5%	628	63.1%		
Some primary	1460	36.1%	256	25.7%		
Some secondary	511	12.6%	63	6.3%		
Secondary or more	397	9.8%	49	4.9%		
HIV status						
HIV−	2800	76.7%	712	78.4%	1.25	.26
HIV +	852	23.3%	196	21.6%		
*Household wealth index*						
Quintile 1 (poorest)	802	19.8%	244	24.2%	12.64	.01*
Quintile 2	799	19.7%	202	20.0%		
Quintile 3	802	19.8%	189	18.8%		
Quintile 4	809	20.0%	198	19.6%		
Quintile 5 (richest)	839	20.7%	175	17.4%		
	Mean ± SD		Mean ± SD		*t*-value	*p*-value
Age	60.5 ± 12.5		66.6 ± 14.2		12.40	< .01**

* *p* < .05.

** *p* < .01

No significant differences were found in sex, HIV status and household wealth with respect to the HIV acquisition risk questions. Significant differences were found in age and education between people who responded versus not responded to the HIV acquisition risk questions; responders were younger and higher educated. With respect to the HIV prevalence questions, responders were not significantly different from non-responders in sex, age and HIV status. However, the groups significantly differed in age, education and wealth status; responders were younger, higher educated, and wealthier

**Table 8:** Poisson regression models to examine the association between perceptional accuracy of HIV acquisition risk and sociodemographic covariates

Covariate	Accurate acquisition risk for man				Accurate acquisition risk for woman			
	Poisson				Poisson			
	RR (95% CI)	*p*-value	RR (95% CI)	*p*-value	RR (95% CI)	*p*-value	RR (95% CI)	*p*-value
*Sex*								
Men	REF		REF		REF		REF	
Women	1.96 (.52–7.40)	.32	1.93 (.50–7.44)	.34	2.53 (.73–8.73)	.14	2.44 (.71–8.40)	.16
*Age groups*								
40–49	REF		REF		REF		REF	
50–59	.28 (.03–2.77)	.27	.28 (.03–2.84)	.28	1.13 (.24–5.45)	.88	1.14 (.23–5.64)	.87
60–69	1.90 (.37–9.79)	.44	1.96 (.36–1.54)	.44	1.88 (.37–9.63)	.45	1.99 (.37–1.57)	.42
70–79	.76 (.07–8.77)	.82	.77 (.06–9.42)	.84	.00 (.00)	1.00	.00 (.00)	1.00
≥ 80	1.85 (.14–23.59)	.64	1.86 (.14–25.46)	.64	3.08 (.37–25.59)	.30	3.45 (.39–3.35)	.27
*Education level*								
No formal education	REF		REF		REF		REF	
Some primary	6.12 (1.18–31.69)	.03*	6.31 (1.19–33.23)	.03*	2.82 (.76–1.53)	.12	2.65 (.70–1.00)	.15
Some secondary	5.53 (.60–51.12)	.13	5.32 (.57–5.03)	.14	3.98(.67–23.53)	.13	3.72(.62–22.38)	.15
Secondary or more	.00 (.00)	1.00	.00 (.00)	1.00	.00 (.00)	1.00	.00 (.00)	1.00
*HIV status*								
HIV−	REF		REF		REF		REF	.16
HIV+	.29 (.04–2.31)	.25	.29 (.04–2.36)	.25	.23 (.03–1.78)	.16	.23 (.30–1.79)	
*Household wealth index*								
Quintile 1 (poorest)	REF		REF		REF		REF	
Quintile 2	1.09 (.18–6.66)	.93	1.06(.17–6.52)	.95	1.43 (.33–6.14)	.63	1.39(.32–5.96)	.66
Quintile 3	.87 (.14–5.51)	.89	.85 (.14–5.31)	.86	.51 (.08–3.19)	.47	.51 (.08–3.15)	.47
Quintile 4	.82 (.13–5.33)	.84	.84(.13–5.50)	.86	.75(.14–4.03)	.73	.75 (.14–4.02)	.73
Quintile 5 (richest)	.00 (.00)	1.00	.00 (.00)	1.00	.26 (.03–2.82)	.27	.26 (.03–2.81)	.27
*Marital status*								
Never married	REF		REF		REF		REF	
Currently married/living with partner	0.33 (0.06–1.81)	.20	.36 (.06–2.04)	.25	.38 (.07–2.02)	.26	.38 (.07–2.03)	.26
Separated/deserted	.00 (.00)	1.00	.00 (.00)	1.00	.26 (.02–2.99)	.28	.26 (.02–3.00)	.28
Divorced	.00 (.00)	1.00	.00 (.00)	1.00	.50 (.04–5.85)	.58	.45 (.04–5.44)	.53
Widowed	.20 (.03–1.50)	.12	.21 (.03–1.54)	.13	.29 (.04–1.88)	.19	.28 (.04–1.86)	.19
*Ever tested for HIV*								
Yes	-	-	REF		-	-	REF	
No	-	-	1.40 (.38–5.10)	.61	-	-	.83 (.25–2.79)	.76

* *p* < .05.

** *p* < .01

**Table 9:** Differences in participant characteristics between underestimates versus overestimates of the HIV prevalence among people 15 and 50 years or older

	HIV prevalence estimate 15 ≥ years					HIV prevalence estimate 50 ≥ years				
	Underestimates		Overestimates		Chi-square	Underestimates		Overestimates		Chi-square
	N	%	N	%	*p*-value	N	%	N	%	*p*-value
*Sex*					.97					.26
Men	57	45.2%	1469	45.4%		92	50.3%	1251	46.0%	
Women	69	54.8%	1764	54.6%		91	49.7%	1470	54.0%	
*Age groups*					.48					.07
40–49	27	21.4%	651	20.1%		37	20.2%	553	20.3%	
50–59	31	24.6%	957	29.6%		48	26.2%	809	29.7%	
60–69	35	27.8%	837	25.9%		59	32.2%	688	25.3%	
70–79	18	14.3%	521	16.1%		19	10.4%	437	16.1%	
≥ 80	15	11.9%	267	8.3%		20	10.9%	234	8.6%	
*Education*					< .01**					.03*
No formal education	72	57.1%	1334	41.3%		88	48.1%	1123	41.3%	
Some primary (1–7 years)	34	27.0%	1165	36.1%		50	27.3%	994	36.6%	
Some secondary (8–11 years)	14	11.1%	409	12.7%		21	11.5%	346	12.7%	
Secondary or more (12 + years)	6	4.8%	320	9.9%		24	13.1%	253	9.3%	
*HIV status*					.94					.07
HIV−	90	76.9%	2230	76.6%		134	82.2%	1865	76.0%	
HIV +	27	23.1%	681	23.4%		29	17.8%	588	24.0%	
*Household wealth index*					.03*					.97
Quintile 1 (poorest)	30	23.8%	637	19.7%		38	20.8%	564	20.7%	
Quintile 2	30	23.8%	659	20.4%		38	20.8%	546	20.1%	
Quintile 3	32	25.4%	630	19.5%		37	20.2%	521	19.1%	
Quintile 4	13	10.3%	643	19.9%		33	18.0%	551	20.2%	
Quintile 5 (richest)	21	16.7%	664	20.5%		37	20.2%	539	19.8%	
*Marital status*					.34					.40
Never married	8	6.3%	185	5.7%		11	6.0%	154	5.7%	
Currently married/living with partner	69	54.8%	1688	52.3%		102	55.7%	1410	51.9%	
Separated/deserted	13	10.3%	250	7.7%		16	8.7%	220	8.1%	
Divorced	2	1.6%	169	5.2%		4	2.2%	144	5.3%	
Widowed	34	27.0%	939	29.1%		50	27.3%	790	29.1%	
Total number of respondents	126		3233			183		2721		

* *p* < .05.

** *p* < .01

**Table 10:** Differences in participant characteristics, sexual risk behavior and testing behavior between accurate reporters, mild overestimators and high overestimators of the HIV prevalence over 15 and 50 years old

	HIV prevalence estimate 15 ≥ years							HIV prevalence estimate 50 ≥ years						
	High overestimators^a^		Mild overestimators^b^		Accurate Reporters^c^		Chi-square	High overestimators^a^		Mild overestimators^b^		Accurate Reporters^c^		Chi-square
	N	%	N	%	N	%	*p*-value	N	%	N	%	N	%	*p*-value
*Sex*							.10							.81
Men	843	45.3%	626	45.6%	349	49.9%		681	46.5%	570	45.3%	530	46.2%	
Women	1018	54.7%	746	54.4%	350	50.1%		782	53.5%	688	54.7%	617	53.8%	
*Age groups*							.32							.33
40–49	386	20.7%	265	19.3%	117	16.7%		296	20.2%	257	20.4%	204	17.8%	
50–59	554	29.8%	403	29.4%	206	29.5%		438	29.9%	371	29.5%	337	29.4%	
60–69	481	25.8%	356	25.9%	210	30.0%		371	25.4%	317	25.2%	334	29.1%	
70–79	288	15.5%	233	17.0%	109	15.6%		233	15.9%	204	16.2%	189	16.5%	
≥ 80	152	8.2%	115	8.4%	57	8.2%		125	8.5%	109	8.7%	83	7.2%	
*Education*							.70							.29
No formal education	787	42.4%	547	39.9%	274	39.2%		635	43.5%	488	38.9%	467	40.7%	
Some primary (1–7 years)	668	36.0%	497	36.3%	264	37.8%		520	35.6%	474	37.8%	416	36.3%	
Some secondary (8–11 years)	227	12.2%	182	13.3%	91	13.0%		178	12.2%	168	13.4%	144	12.6%	
Secondary or more (12+ years)	176	9.5%	144	10.5%	70	10.0%		128	8.8%	125	10.0%	120	10.5%	
HIV status							.01**							.60
HIV−	1249	74.5%	981	79.5%	485	76.7%		998	75.5%	867	76.6%	801	77.3%	
HIV +	428	25.5%	253	20.5%	147	23.3%		323	24.5%	265	23.4%	235	22.7%	
*Household wealth index*							.21							< .01**
Quintile 1 (poorest)	363	19.5%	274	20.0%	134	19.2%		320	21.9%	244	19.4%	200	17.4%	
Quintile 2	392	21.1%	267	19.5%	110	15.7%		312	21.3%	234	18.6%	215	18.7%	
Quintile 3	359	19.3%	271	19.8%	141	20.2%		290	19.8%	231	18.4%	244	21.3%	
Quintile 4	368	19.8%	275	20.0%	159	22.7%		261	17.8%	290	23.1%	225	19.6%	
Quintile 5 (richest)	379	20.4%	285	20.8%	155	22.2%		280	19.1%	259	20.6%	263	22.9%	
*Marital status*							< .01**							< .01**
Never married	107	5.8%	78	5.7%	33	4.7%		98	6.7%	56	4.5%	62	5.4%	
Currently married/living with partner	962	51.7%	726	53.0%	373	53.4%		724	49.6%	686	54.6%	618	53.9%	
Separated/deserted	135	7.3%	115	8.4%	83	11.9%		11	7.7%	107	8.5%	108	9.4%	
Divorced	108	5.8%	60	4.4%	11	1.6%		87	6.0%	57	4.5%	33	2.9%	
Widowed	547	29.4%	392	28.6%	199	28.5%		439	30.0%	351	27.9%	326	28.4%	
*Ever sex without condom with someone HIV positive*							.45							.45
Yes	33	1.8%	21	1.6%	16	2.4%		33	1.8%	21	1.6%	16	2.4%	
No	1806	98.2%	1316	98.4%	659	97.6%		1806	98.2%	1316	98.4%	659	97.6%	
*Sex for money*							.93							.93
Yes	8	.4%	7	.5%	3	.4%		8	.4%	7	.5%	3	.4%	
No	1844	99.6%	1344	99.5%	688	99.6%		1844	99.6%	1344	99.5%	688	99.6%	
*Ever tested for HIV*	1163	62.5%	879	64.2%	543	78.5%	< .01**	1163	62.5%	879	64.2%	543	78.5%	< .01**
Yes	697	37.5%	490	35.8%	149	21.5%		697	37.5%	490	35.8%	149	21.5%	
No														
Total number of respondents	1861		1372		699			1861		1372		699		

* *p*<.05.

** *p*<.01

a High overestimates were defined as people who estimated HIV prevalence to be 57.0% or higher

b Mild overestimates were defined as people who estimated HIV prevalence between 29.9% and 57.0% for prevalence ≥ 15 years old and between 30.0% and 57.0% for prevalence≥50 years old

c Accurate reporters were defined as people who estimated HIV prevalence between 9.9% and 29.9% for prevalenc≥ 15 years old and between 10.0% and 30.0% for prevalence≥ 50 years old

**Table 11:** Differences in participant’ characteristics, sexual risk behavior and testing behavior between accurate reporters, mild overestimators and high overestimators of the HIV acquisition risk for a man and woman

	Acquisition risk for man							Acquisition risk for woman						
	High overestimators^a^		Mild overestimators^b^		Accurate reporters^c^		Chi- square	High overestimators^a^		Mild overestimators^b^		Accurate reporters^c^		Chi- square
	N	%	N	%	N	%	*p*-value	N	%	N	%	N	%	*p*-value
*Sex*							.03*							.03*
Men	1263	44.8%	596	48.9%	4	30.8%		1273	44.8%	581	48.4%	4	25.0%	
Women	1558	55.2%	622	51.1%	9	69.2%		1566	55.2%	620	51.6%	12	75.0%	
*Age groups*							.33							.34
40–49	564	20.0%	228	18.7%	3	23.1%		569	20.0%	220	18.3%	4	25.0%	
50–59	832	29.5%	334	27.4%	1	7.7%		8.35	29.4%	331	27.6%	4	25.0%	
60–69	738	26.2%	344	28.2%	6	46.2%		744	26.2%	337	28.1%	6	37.5%	
70–79	450	16.0%	201	16.5%	1	7.7%		459	16.2%	199	16.6%	0	.0%	
≥ 80	237	8.4%	111	9.1%	2	15.4%		232	8.2%	114	9.5%	2	12.5%	
*Education*							.12							.30
No formal education	1210	42.9%	520	42.7%	3	23.1%		1204	42.5%	526	43.8%	4	25.0%	
Some primary (1–7 years)	975	34.6%	448	36.8%	8	61.5%		998	35.2%	428	35.7%	9	56.3%	
Some secondary (8–11 years)	362	12.8%	128	10.5%	2	15.4%		355	12.5%	134	11.2%	3	18.8%	
Secondary or more (12 + years)	2714	9.6%	121	9.9%	0	.0%		279	9.8%	112	9.3%	0	.0%	
HIV status							.53							.10
HIV−	1957	76.5%	825	76.3%	10	90.9%		1984	77.1%	795	74.6%	13	92.9%	
HIV +	602	23.5%	256	23.7%	1	9.1%		589	22.9%	270	25.4%	1	7.1%	
*Household wealth index*							.06							.05
Quintile 1(poorest)	600	21.3%	214	17.6%	2	15.4%		584	20.6%	229	19.1%	3	18.8%	
Quintile 2	569	20.2%	242	19.9%	3	23.1%		569	20.0%	236	19.7%	6	37.5%	
Quintile 3	537	19.0%	249	20.4%	5	38.5%		564	19.9%	228	19.0%	3	18.8%	
Quintile 4	538	19.1%	263	21.6%	3	23.1%		528	18.6%	278	23.1%	3	18.8%	
Quintile 5 (richest)	577	20.5%	250	20.5%	0	.0%		594	20.9%	230	19.2%	1	6.3%	
*Marital status*							.33							.01*
Never married	52	5.4%	73	6.0%	2	15.4%		155	5.5%	68	5.7	2	12.5%	
Currently married/living with partner	1464	51.9%	632	51.9%	6	46.2%		1480	52.1%	620	51.7	7	43.8%	
Separated/deserted	222	7.9%	114	9.4%	1	7.7%		211	7.4%	124	10.3	2	12.5%	
Divorced	147	5.2%	47	3.9%	0	.0%		153	5.4%	38	3.2	1	6.3%	
Widowed	835	29.6%	351	28.8%	4	30.8%		839	29.6%	350	29.2	4	25.0%	
*Ever sex without condom with someone HIV positive*							.23							.09
Yes	45	1.6%	21	1.8%	1	7.7%		40	1.4%	26	2.2%	1	6.3%	
No	2718	98.4%	1179	98.3%	12	92.3%		2739	98.6%	1158	97.8%	15	93.8%	
*Sex for money*							.70							0.17
Yes	11	.4%	7	.6%	0	.0%		9	.3%	9	.8%	0	.0%	
No	2789	99.6%	1203	99.4%	13	100.0%		2806	99.7%	1187	99.2%	16	100.0%	
*Ever tested for HIV*							.52							0.44
Yes	1855	66.0%	779	64.2%	8	61.5%		1864	65.8%	767	64.2%	12	75.5%	
No	927	34.0%	435	35.8%	5	38.5%		968	34.2%	428	35.8%	4	25.0%	
Total number of respondents	2821		1218		13			2839		1201		16		

* *p* < .05.

** *p* < .01

a High overestimates were defined as people who estimated HIV acquisition risk to be 1 in 1 or 1 in 2

b Mild overestimates were defined as people who estimated HIV acquisition risk to be between 1 in 3 and 1 in 36

c Accurate reporters were defined as people who estimated HIV acquisition risk to be 1 in 37 or less

## References

[R1] Akwara PA, Madise NJ, & Hinde A (2003). Perception of risk of HIV/AIDS and sexual behaviour in Kenya. Journal of Biosocial Science, 35(3), 385–411. 10.1017/S0021932003003857. 12887220

[R2] Barroso PF, Schechter M, Gupta P, Melo MF, Vieira M, Murta FC, Souza Y, & Harrison LH (2000). Effect of antiretroviral therapy on HIV shedding in semen. Annals of Internal Medicine, 133(4), 280–284. 10.7326/0003-4819-133-4-200008150-00039. 10929169

[R3] Belcher L, Strenberg MR, Wolitski RJ, Halikitis P, & Hoff C (2005). Condom use and perceived risk of HIV transmission among sexually active HIV-positive men who have sex with men. AIDS Education and Prevention, 17(1), 79–89. 10.1521/aeap.17.1.79.58690. 15843112

[R4] Boily M, Baggaley RF, Wang L, Masse B, White RG, Hayes RJ, & Alary M (2009). Heterosexual risk of HIV-1 infection per sexual act: Systematic review and meta-analysis of observational studies. The Lancet Infectious Diseases, 9(2), 118–129. 10.1016/S1473-3099(09)70021-0. 19179227 PMC4467783

[R5] Brewer NT, Weinstein ND, Cuite CL, & Herrington JE (2004). Risk perceptions and their relation to risk behavior. Annals of Behavioral Medicine, 27(2), 125–130. 10.1207/s15324796abm2702_7. 15026296

[R6] Chard AN, Metheny N, & Stephenson R (2017). Perceptions of HIV seriousness, risk, and threat among online samples of HIV-negative men who have sex with men in seven countries. JMIR Public Health and Surveillance, 3(2), e37. 10.2196/publichealth.7546. 28634155 PMC5497068

[R7] Clifton S, Nardone A, Field N, Mercer CH, Tanton C, Macdowall W, Johnson AM, & Sonnenberg P (2016). HIV testing, risk perception, and behaviour in the British population. AIDS, 30(6), 943–952. 10.1097/QAD.0000000000001006. 26963528 PMC4794135

[R8] Cohen MS, Chen YQ, McCauley M, Gamble T, Hosseinipour MC, Kumarasamy N, Hakim JG, Kumwenda J, Grinsztejn B, Pilotto JHS, Godbole SV, Chariyalertsak S, Santos BR, Mayer KH, Hoffman IF, Eshleman SH, Piwowar-Manning E, Cottle L, Zhang XC, … Fleming TR (2016). Antiretroviral therapy for the prevention of HIV-1 transmission. New England Journal of Medicine, 375(9), 830–839. 10.1056/NEJMoa1600693. PMC5049503 27424812

[R9] Filmer D, & Pritchett LH (2001). Estimating wealth effects without expenditure data–or tears: An application to educational enrollments in states of India. Demography, 38(1), 115–132. 10.1353/dem.2001.0003. 11227840

[R10] Geldsetzer P, Vaikath M, Wagner R, Rohr JK, Montana L, Gómez-Olivé FX, Rosenberg MS, Manne-Goehler J, Mateen FJ, Payne CF, Kahn K, Tollman SM, Salomon JA, Gaziano TA, Bärnighausen T, & Berkman LF (2018). Depressive symptoms and their relation to age and chronic diseases among middle-aged and older adults in rural South Africa. Journals of Gerontology Series A, 74, 957–963. 10.1093/gerona/gly145. PMC6521913 29939214

[R11] Gerrard M, Gibbons FX, & Bushman BJ (1996). Relation between perceived vulnerability to HIV and precautionary sexual behavior. Psychological Bulletin, 119(3), 390–409. 10.1037/0033-2909.119.3.390. 8668745

[R12] Gerrard M, Gibbons FX, Warner TD, & Smith GE (1993). Perceived vulnerability to HIV infection and AIDS preventive behavior: A critical review of the evidence. In Pryor JB & Reeder GD (Eds.), The social psychology of HIV infection (pp. 59–84). Lawrence Erlbaum Associates, Inc. https://psycnet.apa.org/record/1995-98031-003

[R13] GHO∣Number of new HIV infections - Data by WHO region. (2018). In World Health Organization (WHO). http://apps.who.int/gho/data/view.main.HIVINCIDENCEREGIONv?lang=en

[R14] Gómez-Olivé FX, Angotti N, Houle B, Klipstein-Grobusch K, Kabudula C, Menken J, Williams J, Tollman S, & Clark SJ (2013). Prevalence of HIV among those 15 and older in rural South Africa. AIDS Care, 25(9), 1122–1128. 10.1080/09540121.2012.750710. 23311396 PMC3778517

[R15] Gómez-Olivé FX, Montana L, Wagner RG, Kabudula CW, Rohr JK, Kahn K, Bärnighausen T, Collinson M, Canning D, Gaziano T, Salomon JA, Payne CF, Wade A, Tollman SM, & Berkman L (2018). Cohort profile: Health and ageing in Africa: A longitudinal study of an INDEPTH community in South Africa (HAALSI). International Journal of Epidemiology, 47(3), 689–690. 10.1093/ije/dyx247. 29325152 PMC6005147

[R16] Halkitis PN, Zade DD, Shrem M, & Marmor M (2004). Beliefs about HIV non-infection and risky sexual behavior among MSM. AIDS Education and Prevention, 16(5), 448–458. 10.1521/aeap.16.5.448.48739. 15491956

[R17] Hess RF, & Mbavu M (2010). HIV/AIDS fatalism, beliefs and prevention indicators in Gabon: Comparisons between Gabonese and Malians. African Journal of AIDS Research, 9(2), 125–133. 10.2989/16085906.2010.517479. 25860521

[R18] Hollingsworth TD, Anderson RM, & Fraser C (2008). HIV-1 Transmission, by stage of infection. Journal of Infectious Diseases, 198(5), 687–693. 10.1086/590501. 18662132

[R19] Kahn K, Collinson MA, Gómez-Olivé FX, Mokoena O, Twine R, Mee P, Afolabi SA, Clark BD, Kabudula CW, Khosa A, Khoza S, Shabangu MG, Silaule B, Tibane JB, Wagner RG, Garenne ML, Clark SJ, & Tollman SM (2012). Profile: Agincourt health and socio-demographic surveillance system. International Journal of Epidemiology, 41(4), 988–1001. 10.1093/ije/dys115. 22933647 PMC3429877

[R20] Kalichman SC, & Cain D (2005). Perceptions of local HIV/AIDS prevalence and risks for HIV/AIDS and other sexually transmitted infections: Preliminary study of intuitive epidemiology. Annals of Behavioral Medicine, 29(2), 100–105. 10.1207/s15324796abm2902_4. 15823783

[R21] Kalichman SC, Eaton L, Cain D, Cherry C, Pope H, & Kalichman M (2006). HIV treatment beliefs and sexual transmission risk behaviors among HIV positive men and women. Journal of Behavioral Medicine, 29(5), 401–410. 10.1007/s10865-006-9066-3. 16944306

[R22] McKeever M (2017). Educational inequality in apartheid South Africa. American Behavioral Scientist, 61(1), 114–131. 10.1177/0002764216682988.

[R23] Meyer-Weitz A (2005). Understanding fatalism in HIV/AIDS protection: The individual in dialogue with contextual factors. African Journal of AIDS Research, 4(2), 75–82. 10.2989/16085900509490345. 25870883

[R24] Miller WC, Rosenberg NE, Rutstein SE, & Powers KA (2010). Role of acute and early HIV infection in the sexual transmission of HIV. Current Opinion in HIV and AIDS, 5(4), 277–282. 10.1097/COH.0b013e32833a0d3a. 20543601 PMC3130067

[R25] Mills EJ, Bärnighausen T, & Negin J (2012). HIV and aging—preparing for the challenges ahead. New England Journal of Medicine, 366(14), 1270–1273. 10.1056/NEJMp1113643. 22475591

[R26] Mills EJ, Rammohan A, & Awofeso N (2011). Ageing faster with AIDS in Africa. Lancet, 377(9772), 1131–1133. 10.1016/S0140-6736(10)62180-0. 21126759

[R27] Negin J, Bärnighausen T, Lundgren JD, & Mills EJ (2012). Aging with HIV in Africa: The challenges of living longer. AIDS, 26, S1–S5. 10.1097/QAD.0b013e3283560f54. 22713477 PMC4017661

[R28] Noroozinejad G, Yarmohmmadi Vasel M, Bazrafkan F, Sehat M, Rezazadeh M, & Ahmadi K (2013). Perceived risk modifies the effect of HIV knowledge on sexual risk behaviors. Frontiers in Public Health, 1, 33. 10.3389/fpubh.2013.00033. 24350202 PMC3860014

[R29] Parker R, & Aggleton P (2003). HIV and AIDS-related stigma and discrimination: A conceptual framework and implications for action. Social Science and Medicine, 57(1), 13–24. 10.1016/S0277-9536(02)00304-0. 12753813

[R30] Patel P, Borkowf CB, Brooks JT, Lasry A, Lansky A, & Mermin J (2014). Estimating per-act HIV transmission risk: A systematic review. AIDS, 28(10), 1509–1519. 10.1097/QAD.0000000000000298. 24809629 PMC6195215

[R31] Price JT, Rosenberg NE, Vansia D, Phanga T, Bhushan NL, Maseko B, Brar SK, Hosseinipour MC, Tang JH, Bekker L-G, & Pettifor A (2018). Predictors of HIV, HIV risk perception, and HIV worry among adolescent girls and young women in Lilongwe, Malawi. Journal of Acquired Immune Deficiency Syndromes, 77(1), 53–63. 10.1097/QAI.0000000000001567. 28991885 PMC5720919

[R32] Rammohan A, & Awofeso N (2010). Addressing HIV prevention and disease burden among Africans aged over 50 years. Tropical Doctor, 40(3), 171–172. 10.1258/td.2010.090492. 20555048

[R33] Rodger AJ, Cambiano V, Bruun T, Vernazza P, Collins S, van Lunzen J, Corbelli GM, Estrada V, Geretti AM, Beloukas A, Asboe D, Viciana P, Gutiérrez F, Clotet B, Pradier C, Gerstoft J, Weber R, Westling K, Wandeler G, Lundgren J (2016). Sexual activity without condoms and risk of HIV transmission in serodifferent couples when the HIV-positive partner is using suppressive antiretroviral therapy. Journal of the American Medical Association, 316(2), 171–181. 10.1001/jama.2016.5148. 27404185

[R34] Rosenberg ES, Grey JA, Sanchez TH, & Sullivan PS (2016). Rates of prevalent HIV infection, prevalent diagnoses, and new diagnoses among men who have sex with men in US states, metropolitan statistical areas, and counties, 2012–2013. JMIR Public Health and Surveillance, 2(1), e22. 10.2196/publichealth.5684. 27244769 PMC4887662

[R35] Rosenberg MS, Gómez-Olivé FX, Rohr JK, Houle BC, Kabudula CW, Wagner RG, Salomon JA, Kahn K, Berkman LF, Tollman SM, & Bärnighausen T (2017). Sexual behaviors and HIV status: A population-based study among older adults in rural South Africa. Journal of Acquired Immune Deficiency Syndromes, 74(1), e9–e17. 10.1097/QAI.0000000000001173. 27926667 PMC5147032

[R36] Sandelowski M, Lambe C, & Barroso J (2004). Stigma in HIV-positive women. Journal of Nursing Scholarship, 36(2), 122–128. 10.1111/j.1547-5069.2004.04024.x. 15227758

[R37] Sileo KM, Bogart LM, Wagner GJ, Musoke W, Naigino R, Mukasa B, & Wanyenze RK (2019). HIV fatalism and engagement in transactional sex among Ugandan fisherfolk living with HIV. SAHARA J: Journal of Social Aspects of HIV/AIDS Research Alliance, 16(1), 1–9. 10.1080/17290376.2019.1572533. PMC6366790 30727838

[R38] Sterck O (2013). HIV/AIDS and fatalism: should prevention campaigns disclose the transmission rate of HIV? Journal of African Economies, 23(1), 53–104. 10.1093/jae/ejt018.

[R39] Treves-Kagan S, El Ayadi AM, Pettifor A, MacPhail C, Twine R, Maman S, Peacock D, Kahn K, & Lippman SA (2017). Gender, HIV testing and stigma: The association of HIV testing behaviors and community-level and individual-level stigma in rural South Africa differ for men and women. AIDS and Behavior, 21(9), 2579–2588. 10.1007/s10461-016-1671-8. 28058565 PMC5498263

[R40] UNFPA. (2015). Emerging evidence, lessons and practice comprehensive sexuality education: A global review. United Nations Educational, Scientific and Cultural Organization (UNESCO). https://www.unfpa.org/sites/default/files/pub-pdf/CSE_Global_Review_2015.pdf

[R41] van Heerden A, Barnabas RV, Norris SA, Micklesfield LK, van Rooyen H, & Celum C (2017). High prevalence of HIV and non-communicable disease (NCD) risk factors in rural KwaZulu-Natal, South Africa. Journal of the International AIDS Society, 20(2), e25012. 10.1002/JIA2.25012. PMC5810314 29064168

[R42] White D, & Stephenson R (2016). Correlates of perceived HIV prevalence and associations with HIV testing behavior among men who have sex with men in the United States. American Journal of Men’s Health, 10(2), 90–99. 10.1177/1557988314556672. 25389216

[R43] World Health Organization (WHO). (2019). South Africa - HIV Country Profile 2019. https://cfs.hivci.org/country-factsheet.html

